# Spillover effect of workplace democracy: A conceptual revision

**DOI:** 10.3389/fpsyg.2022.933263

**Published:** 2022-12-08

**Authors:** Irma Rybnikova

**Affiliations:** Department Lippstadt 2, Hamm-Lippstadt University of Applied Sciences, Hamm, Germany

**Keywords:** workplace democracy, political participation, spillover effect, Pateman, conceptual revision

## Abstract

The so-called “spillover thesis” by Pateman is one of the prominent theoretical explanations for why workplace-based participation and democracy could induce stronger political participation. By this thesis, Pateman underscored the relevance of industrial workplaces as relevant places where citizens can be socialized regarding democratic attitudes while proposing the educative effect of workplace democracy and assuming a strong linkage between workplace-based and political participation as moderated by self-efficacy. The spillover thesis has received a controversial consideration as previous empirical studies have provided inconsistent evidence. Some empirical undertakings support the assumption by Pateman and indicate a positive relationship between workplace democracy and societal effects, like increased moral and community orientation or higher levels of political participation among employees from companies with higher degrees of workplace democracy. Other empirical studies yield results that do not confirm the thesis. Scholars have discussed *method-based* shortcomings of the previous empirical research while pointing to the inconsistency of definitions and operationalizations as the main shortcoming. In contrast to that, systematic *conceptual* consideration of the spillover thesis and the accompanying scholarship are still lacking. The present article addresses this shortcoming and provides a critical reflection on the spillover thesis and corresponding research. It aims at identifying the main conceptual shortcomings and providing avenues for future theoretical undertakings in analyzing whether and how participation at the workplace is related to participation in political domains.

## Introduction

Although democracy is one of the contested terrains of social societies, the relationship between political democracy and workplace democracy represents a relatively understudied issue of political sciences as well as organizational sociology and psychology. In 1916, John Dewey pointed out, in his seminal work “Democracy and Education,” that democracy in society is an educative project. According to him, schools could be considered hotbeds of democratic society since democratic practices can be learned there. While proposing the genuine nexus between the educational realm and the development of democracy, Dewey (1916/1997) suggested that democratic skills could be acquired in institutional settings beyond the mere political context.

Currently, in many European democratic societies, widespread disillusionment and apathy regarding democratic systems could be observed; in some of those, oligarchic tendencies are becoming evident. The unsettling diagnosis, as formulated in the debate on post-democracy (Crouch, 2004), is seemingly becoming true: Small economic elites seem to dominate political decisions, whereas political parties and their leaders are largely concerned with marketing strategies and tools for vote generation. Given this diagnosis, the question whether political participation and genuine interest in democratic mechanisms could be nurtured by workplace democracy becomes particularly important.

In the field of industrial relations, it has been widely acknowledged that employment and its quality could have relevant social consequences. Scholars pointed to the nexus between workplaces and social domains by using the metaphor “the long arm of the job” (e.g., Meissner, 1971). One of the research areas that are particularly informative for dealing with this issue is the literature on workplace democracy. It includes research that spans different disciplines, including industrial relations, management and organization studies, organizational sociology, and psychology, and is related to different forms of work autonomy and organizational democracy.

When it comes to the question whether workplace democracy is related to political participation, scholars frequently refer to the so-called “spillover hypothesis” by Pateman (1970) as the conceptual foundation. The thesis points to the general educative effect of employee participation at the workplace, which is assumed to lead to higher political efficacy and civic attitudes among employees. Over time, employees are supposed to take part in political processes beyond the workplace in a way that is in line with democratic ideas. Pateman (1970) extended the focus beyond educative systems, as argued by Dewey (1916/1997), and supposed that business companies and workplaces bear an important educative effect of democracy.

The vast majority of the research on workplace democracy has focused exclusively on the analysis of individual or organizational effects such as employee satisfaction, company loyalty, innovative behaviors, or financial performance of the company (e.g., Long, 1982; McNabb and Whitfield, 1998; Cox et al., 2006). The extent to which workplace democracy can make a contribution beyond the focal company, that is, to society and politics by strengthening political democracy has received relatively little attention from scholars. Moreover, existing empirical studies (e.g., Adams, 1992; Weber et al., 2009; Weber and Unterrainer, 2015; Budd et al., 2018; Timming and Summers, 2020; Weber et al., 2020) have yielded inconsistent results. The meta-analysis performed by Weber et al. (2020) indicated that employees from companies with higher degrees of workplace democracy tend toward an increased moral and community orientation and higher levels of political participation than individuals from organizations with lesser or no democratic arrangements. From the methodological perspective, empirical undertakings in this field have been criticized because of measurement problems, like inconsistent operationalization of dependent and independent variables (e.g., Kim, 2021). Until now, a research synthesis and a critical conceptual reflection of the studies undertaken in regard to democratic spillover from workplace contexts into political settings have only occurred in a fragmented way and thus calls for a more systematic and comprehensive approach.

Thus, the present article is a conceptual contribution that seeks to challenge the existing debate regarding the relationship between workplace democracy and its political effects by, first, identifying limitations and blind spots of previous conceptual discussion in this field and, second, by figuring out avenues for how our thinking of the nexus between the workplace-based democracy and political participation could be improved and could be studied empirically in prospective research.

While seeking to critically summarize and redirect existing research on democratic spillover, the present study rests on *an integrative literature review* as a method tool (e.g., Cronin and George, 2020). Empirical and conceptual articles that explicitly address the democratic spillover, according to Pateman (1970), were gathered through the search in academic databases, extended by specific references on the side of colleagues. The resulting research stems from various sub-disciplines of social sciences and respective communities of practice, including industrial sociologists, organizational psychologists, and management scholars. As the topic of this conceptual synthesis is the democratic spillover, the focus was solely given to the respective literature. In order to keep the scope of the article consistent, research that indirectly relates to the field of workplace democracy, for example, studies on participative leadership in organizations, has been omitted from the analysis as long as it does not explicitly deal with societal effects. All gathered articles have been juxtaposed and analyzed thematically regarding the main empirical and conceptual issues, like the operationalization of dependent and independent variables or conceptual frameworks used. The final list of the articles considered is provided in Table 1.

**Table 1:** Overview of empirical studies addressing democratic spillover.

	**Authors**	**Research context**	**Variables**	**Method**	**Results**
1	Adman, 2008	Sweden	*Independent variables:* Workplace influence and participation (e.g., job autonomy in terms of perceived influence over the work conditions, face-to-face participation in terms of taking part in decision-making at work, workplace skill acts in terms of preparing meetings, holding presentations or writing letters)	Quantitative panel survey	No significant effect in long-term consideration
			*Dependent variable(s):* Political participation (e.g., voting, membership in a political party, signing a petition)		
2	Budd et al., 2018	27 EU-countries	*Independent variables:* Employee participation (e.g., taking part in strategic decisions or decision about when to start and to finish work)	Quantitative survey (European Social Survey)	Support for a democratic spillover
			*Dependent variable(s):* Political participation (e.g., voting, membership in a political party, signing a petition)		
3	Elden, 1981	USA; a non-union company	*Independent variables:* Job autonomy; beliefs regarding equity in decision making	Quantitative case-study	A significant relationship between job autonomy, political efficacy, and social participation
			*Dependent variable(s):* Feelings of political efficacy; personal potency; social participation		
4	Geurkink et al., 2020	The Netherlands	*Independent variables:* Responses of supervisors to employee voices (suppressive vs. supportive)	Quantitative survey	A negative spillover between work and politics: suppressive responses of supervisors to employee voices trigger political participation
			*Dependent variable(s):* Political participation (e.g., voting, membership in a political party, signing a petition)		
5	Greenberg, 1986	USA; employee-owned companies contrasted with traditional companies	*Independent variables:* Workplace participation ( i.e., being an employed member of a cooperative or not)	Case studies based on qualitative interviews and quantitative survey (cross-sectional and long-term)	Limited and mixed effects of workplace participation on dependent variables; workers in cooperatives are not more politically efficacious than employees of conventional firms and only slightly more participating in politics
			*Dependent variable(s):* Dealienation; democratic citizenship; class consciousness		
6	Greenberg et al., 1996	Democratic (e.g., cooperatives, employee stock ownership) vs. conventional union and non-union enterprises (wood mills)	*Independent variables:* Job autonomy index including participation in decision making, voting for board members, representative participation	Quantitative survey	Political participation is lower in democratic plants than in conventional plants; a negative relationship between workplace democracy and participation in voting; different forms of workplace democracy are related to different indicators of political participation, e.g., autonomy on the job is linked to participation in community affairs but not to voting or political campaigns; representative participation is negatively associated with all three forms of political participation
			*Dependent variable(s):* Participation in voting, participation in campaign activities participation in community affairs		
7	Jian and Jeffres, 2008	USA	*Independent variables:* Decision involvement at work; job autonomy; work community participation	Quantitative survey	Partial support of the democratic spillover: Decision involvement at work is positively associated with political voting; work community participation is positively associated with involvement in local communities, political parties, and campaign activities
			*Dependent variable(s):* Political participation (e.g., voting, membership in a political party, signing a petition)		
8	Lopes et al., 2014	15 EU countries	*Independent variables:* Autonomy at work (e.g., ability to change work methods, speed rate, independency from direct control, ability to learn new things)	Quantitative panel survey (European Working Conditions Survey)	There is a positive link between work autonomy and all indicators of civic behavior
			*Dependent variable(s):* Civic behavior (e.g., membership in political parties, trade unions, or volunteer work)		
9	Madsen, 1997	Denmark; trade union members	*Independent variables:* Work organization, e.g., self-governing groups, on-the-job-liberties; personal development and self-management at work; workplace participation in terms of workplace meetings, after-work meetings, having elected posts	Quantitative survey	The link between workplace democracy and political participation is moderated by employees' orientations: the spillover effect proves valid for collectivistic, but not for individualistic-oriented employees
			*Dependent variable(s):* Political participation (e.g., voting, membership in a political party, signing a petition)		
10	Mays, 2018	Germany	*Independent variables:* Autonomy at work (i.e., supervisory job, flexible working hours, degree of professional autonomy)	Longitudinal panel survey (SOEP)	Partial support for the democratic spillover: For the period 1985-2001, there is a link between some indications of autonomy at work (esp. having a supervisory position) and political interest as well as civic engagement
			*Dependent variable(s):* Political interest; political participation (e.g., voting, membership in a political party, signing a petition)		
11	Peterson, 1992	USA; residents of a small city	*Independent variables:* Workplace politicization (e.g., workplace as politics, workplace efficacy, workplace participation, i.e., taking part in workplace decisions)	Quantitative survey	A significant positive relationship between workplace politicization (all elements considered) and political participation
			*Dependent variable(s):* Political orientations (e.g., political interest and efficacy); political participation (e.g., voting, membership in a political party, signing a petition)		
12	Pircher Verdorfer et al., 2013	North Italy; cooperatives and conventional firms compared	*Independent variables:* Organizational democracy (i.e., working in a cooperative)	Quantitative survey	Organizational democracy has a positive impact on the perceived socio-moral climate in organizations which is positively related to pro-social work-related employees' attitudes
			*Dependent variable(s):* Perceived socio-moral climate (e.g., support and care by supervisors and colleagues; organizational concern for the individual); pro-social and community-related behavioral orientations (e.g., altruism, solidarity at work, democratic engagement orientation)		
13	Sobel, 1993	USA	*Independent variables:* Occupational involvement, e.g., authority in terms of formal ability to instruct others, supervisory responsibility, work participation in terms of involvement in workplace decisions, job participation in terms of participation in running own job	Quantitative survey	Partial support for the democratic spillover: occupational involvement leads to political participation
			*Dependent variable(s):* Political participation (e.g., voting, membership in a political party, signing a petition)		
14	Spreitzer, 2007	32 countries	*Independent variables:* Participative leadership; empowerment at work in terms of decision-making freedom	Quantitative secondary analysis from the GLOBE project and European Value Survey	The relationship between participative leadership, employee empowerment at work, and peace is moderated by the legitimacy of leadership style, reduced feelings of helplessness, and building capability for voice
			*Dependent variable(s):* Peace in terms of the perceived level of corruption within a country, risk of political instability, armed conflict, social unrest, and international disputes and tensions		
15	Tak, 2017	Venezuela; cooperative members compared with members from other associations	*Independent variables:* Frequency of attending membership meetings	Quantitative survey	Cooperative members have a higher likelihood of being involved in community matters than those from other types of associations
			*Dependent variable(s):* Community involvement (e.g., donating money, attending community meetings, trying to organize groups to solve specific problems)		
16	Timming and Summers, 2020	27 EU-countries	*Independent variables:* Participation in decision-making (i.e., perceived ability to decide how the work is organized, to influence workplace decisions and work pace)	Quantitative survey (European Social Survey)	Participation in decision-making is strongly linked to an increased interest in politics and wider pro-democracy affect
			*Dependent variable(s):* Interest in politics; pro-democracy affect		
17	Weber et al., 2008	Austria, North Italy, South Germany, and Liechtenstein; employees from small and medium-sized enterprises	*Independent variables:* Organizational democracy (i.e., different forms of structurally embedded workers‘participation in decision making including co-determination; seven levels of organizational democracy)	Quantitative survey	Democratic structures lead to a higher level of socio-moral atmosphere which positively influences employees' solidarity and the willingness to engage in democratic undertakings
			*Dependent variable(s):* Socio-moral atmosphere; work-related pro-social orientations (e.g., empathy, solidarity at work); community-related value orientations (e.g., willingness to act socially responsible)		
18	Weber et al., 2009	Austria, North Italy, and Southern Germany	*Independent variables:* Perceived participation in decision-making	Quantitative survey	Workplace democracy is positively related to the socio-moral climate, organizational commitment, and prosocial and community-related behavioral orientations of employees
			*Dependent variable(s):* Perceptions of socio-moral climate; pro-social behavioral orientations; democratic values; commitment to the firm		
19	Weber et al., 2020	Democratic enterprises; national and international samples	*Independent variables:* Organizational democracy (i.e., participation structures, employee ownership, perceived participation in organizational decision-making) *Dependent variable(s):* Psychological outcomes (e.g., job satisfaction, job involvement/work motivation); prosocial work behaviors; civic and democratic work behaviors; perceived supportive organizational climate	Meta-analysis	Employees' perceived participation in decision-making is related to pro-social and civic behavioral orientations; employees' direct participation in strategic and tactical organizational decisions are related to value-based commitment, job involvement, and job satisfaction as well as to a supportive climate
					

Since, Kim (2021) provided a comprehensive literature review on methodological shortcomings of the literature on democratic spillover, the main focus in the present analysis is given to the conceptual features of the selected literature, for example, the way the democratic spillover is conceptualized, different mechanisms of democratic spillover, and mainly neglected alternative explanations.

Before we proceed with the analysis of the debate on democratic spillover, one more comment is needed. There is a long-lasting controversy regarding suitable terminology. Some scholars more or less strictly discerned “organizational/workplace democracy” from “employee participation,” arguing that employee participation is a preliminary form of workplace democracy that is usually arranged by managers and mainly relates to operational issues (Weber, 1999). In contrast to this, workplace democracy is said to refer to structural instances of co-determination and strategic co-decision-making by employees or organizational members. Other authors (e.g., Dachler and Wilpert, 1978) did not distinguish between these terms so strictly, arguing that they are strongly interlinked and there exist only slight or gradual differences between them Wegge et al. (2010) provided an integrative approach toward the conceptualization of workplace democracy, which encompasses institutional (e.g., employee-managed organizations), organizational (e.g., employee participation in decision-making, works councils), and group-related (e.g., shared leadership) levels of workplace democracy (Wegge et al., 2010; p. 155). Following the latter and for the aims of consistency, the term “employee participation” will be abandoned in the following text, and the terms workplace and organizational democracy will be used synonymously for depicting any institutional arrangements that aim at providing employees with opportunities to take part in workplace-related decision-making.

The argument of the present conceptual analysis will proceed as follows: After delineating the argument on democratic spillover, as made by Pateman (1970), I summarize the main results and limitations of previous empirical studies that have tried to verify Pateman's thesis. In the main section of the article, I discuss the most relevant conceptual limitations of the previous debate, like different mechanisms of the democratic spillover, different potential kinds of this relationship, and the multidetermined nature of the considered link. The last section of the article includes several conceptual and methodological avenues for prospective research in this field.

## The “spillover hypothesis” by Carole Pateman

Although based in political sciences, Carole Pateman, a British-American scholar, is one of the few authors who are referred to in organizational psychology and sociology when it comes to conceptual underpinnings of workplace democracy as she provides one explanation for how organizational and societal layers of democracy could be linked. Nevertheless, Pateman's argument is driven by the democracy debate in political sciences. In “Participation and Democratic Theory” (1970), one of her earlier works, Pateman differentiated between two concepts of democracy. The first concept is the “elitist” understanding of democracy, which is based on the elite power of a few due to the apathy of voters. In contrast to it, Pateman proposed an alternative, “participative” understanding of democracy. This concept draws on wider participation by the masses, pointing to the fact that the apathy of voters is not the premise of democracy but the result of an elitist approach that limits participation opportunities to few individuals.

The argument developed by Pateman rests on two major assumptions. The first goes back to Jean-Jacques Rousseau's political theory. Based on that, Pateman assumed that individuals and social institutions “cannot be considered in isolation” (Pateman, 1970; p. 42). As psychological attitudes of individuals are strongly interlinked with authority structures in institutions individuals are encountered with, these institutions could have educative effects and frame individual attitudes in favor of democratic developments. Accordingly, Pateman argued that “the major function of participation is an educative one” (Pateman, 1970, p. 38). Together with Rousseau, Pateman based her argument on the wide understanding of “education” that refers to developing “responsible, individual social and political action,” during which individuals learn to take “wider matters than their own immediate private interest” into account and to learn “that the private and public interests are interlinked” (Pateman, 1970, p. 24–25). Pateman summarized that “participation in the workplace” or “industrial democracy” (Pateman, 1970, p. 54) bears the humanistic potential of personality development toward a democratic personality. This kind of education takes place through the psychological mechanism of increased political efficacy as well as through gaining practice in political skills and procedures (Pateman, 1970, p. 42).

In her second major proposition, Pateman has dealt with the role of immediate workplaces while developing democratic personalities. Here, she referred to George D.H. Cole and John Stuart Mill, both of whom argued that democratic principles can be applied beyond the purely political sphere. Among others, labor structures and workplaces could be approached from the perspective of democracy and participation since here, issues such as task differentiation and collective task accomplishment, subordination, and superiority stand at the core of social activity. Because of that, workplaces represent suitable settings for the educative effects of participation. Furthermore, Pateman pointed out that democracy requires a wide net of “social training” toward democratic attitudes and psychological qualities that take part in workplaces through the process of everyday participation. To be educative in democratic terms, workplaces should entail democratic or participative elements. Pateman stressed the utmost relevance of democratization of the authority structures in industry. According to the author, individuals who are able to exercise a certain amount of control “over their job and job environment” and are participating in decision-making in their companies can develop attitudes and qualities as required by the democratic processes (Pateman, 1970, p. 56). Pateman suggested enabling such participative structures at workplaces in order to establish a basis for a democratic society (Pateman, 1970, p. 43). By democratizing industrial settings and by providing equal participation to ordinary people, the author said that the reasons for economic and political inequality could be diminished too.

To sum up, Pateman claimed that industry and work organizations play a crucial role in socializing employees toward democracy through participative workplaces (Pateman, 1970, p. 42–43). Hence, workplace democracy is supposed to have an educative function in democratic terms, and the industry is assumed to play a crucial role in sustaining a democratic society (Pateman, 1970, p. 44).

Although her first proposition is based on a genuine psychological effect, Pateman did not explicate the role of underlying psychological processes sufficiently. According to the author, “experiences of participation in some way leaves the individual better psychologically equipped to undertake further participation in the future” (Pateman, 1970, p. 45). Participation may induce “the sense of political efficacy and political competence” (Pateman, 1970, p. 46). While quoting some psychologists of her time, Pateman argues that the sense of political efficacy is related to personal effectiveness and belief in one's competence, which leads people to be more likely to participate in politics (Pateman, 1970, p. 46). Based on selected empirical studies at the time of her writing this article, Pateman could see evidence supporting her theory and confirming that participation in different spheres is necessary to develop political efficacy and that industry represents the most important sphere for participative education (Pateman, 1970, p. 50–21).

Two notes are relevant concerning the terminology used in the debate. First, Pateman does not use the term “spillover” in her work. It could be an intriguing undertaking to figure out how the original thesis of democratic socialization became “the spillover hypothesis” in the research literature. Second, it is relevant to mention that “spillover” represents a metaphor describing the processes mentioned. However, this metaphor is not completely adequate for what Pateman proposes since it implies a rather mechanistic view of participation. The metaphor of a spillover frames participation as a liquid that cumulates and spills over from one barrel (sphere) into the next as soon as a critical point is achieved. In contrast to that, Pateman's argument represents in its core a “socialization and learning hypothesis,” pointing to the fact that people become socialized or learn democratic and participative processes in different domains (e.g., education, work, and politics) through accumulating skills and qualities, like political efficacy, as needed for political participation.

## Spillover thesis: Empirical and method-based issues

### Mixed empirical findings from previous research

For many decades, workplace democracy has attracted the attention of scholars and researchers from different subfields of social sciences, including organizational psychology. One of the questions workplace democracy research traditionally deals with is the issue of the consequences of democratic and participative schemes in companies. The vast majority of existing research has focused on organizational and thus company-based consequences, either in terms of psychological performance, such as employee satisfaction, motivation, loyalty, and innovative behaviors (e.g., Jenkins and Lawler, 1981; Miller and Monge, 1986; Geralis and Terziovski, 2003; Bakan et al., 2004; Wright and Kim, 2004; Pereira and Osburn, 2007), or in terms of financial performance (e.g., Long, 1982; McNabb and Whitfield, 1998; Cox et al., 2006).

Although mentioned several times (e.g., Dachler and Wilpert, 1978; Adams, 1992), the extent to which workplace democracy can contribute to society by strengthening processes of political democracy has been addressed considerably less, with the result that empirical findings are relatively scant. In his critical overview, Kim (2021) identified 25 studies published in international journals between 1981 and 2020, where scholars have explicitly dealt with the relationship between workplace democracy and political participation. Empirical findings regarding the spillover effect have been heterogeneous and contradicting so far.

The majority of empirical findings from previous studies (Elden, 1981; Peterson, 1992; Madsen, 1997; Spreitzer, 2007; Weber et al., 2008, 2009; Pircher Verdorfer et al., 2013; Lopes et al., 2014; Tak, 2017; Budd et al., 2018; Timming and Summers, 2020) has indicated a more or less significant relationship between workplace democracy and political activities. This result proves true despite the highly diverse research settings and contexts that are encountered here. Some studies are based on cross-sectional data from one particular country, like the United States, Italy, Denmark, or Venezuela (Elden, 1981; Peterson, 1992; Madsen, 1997; Pircher Verdorfer et al., 2013; Tak, 2017); other studies are conducted by drawing on international panel data (Spreitzer, 2007; Lopes et al., 2014; Budd et al., 2018; Timming and Summers, 2020). Some work particularly has focused on cooperatives and employee-owned organizations (e.g., Weber et al., 2008; Pircher Verdorfer et al., 2013; Tak, 2017) while assuming a dense workplace democracy there; the remaining work has not taken this specific form of organizations into account.

However, there is also empirical work that has yielded only partial or limited empirical proof (Sobel, 1993; Jian and Jeffres, 2008; Mays, 2018; Weber et al., 2020). Studies performed by Greenberg (1986), Greenberg et al. (1996), and Adman (2008) have shown that the relationship between workplace democracy and political participation is either insignificant or inconclusive or there is no link. Moreover, Geurkink et al. (2020) observed an oppositional relationship in their study, with political participation being triggered not because of democratic workplaces but also because democratic instances at work are lacking or opportunities for workplace democracy and voice expression are suppressed by supervisors. This shows that not workplace democracy but its prevention leads to political activities, such as voting or becoming a member of a political party.

### Method-based debates

Even though there are a number of empirical studies that provide at least partial support for a spillover effect from the workplace context into the political sphere, definitive empirical evidence of the conceptual argument by Pateman is still weak and not sufficient. Potential reasons for that have been seen in several methodological shortcomings of the empirical undertakings so far. In a self-critical manner, authors have mentioned a number of methodological challenges, like endogeneity, selection biases, generally omitted variable biases, and reverse causality (Budd et al., 2018). In his analysis of measurement issues in the previous empirical research regarding democratic spillover, Kim (2021) identified several shortcomings that limit the validity of previous empirical research. In particular, Kim (2021) pointed *to heterogeneous construct operationalizations*, which lead to inconsistent and mainly insufficient measures as used by the scholars in the case of the independent variable (i.e., workplace democracy) and dependent variable (i.e., political activity). In the following paragraphs, I deal with both variables and respective constructs in more detail.

*Workplace democracy as the independent variable* is a particularly complex terrain. This has to do a lot with the research history. The concept of workplace democracy goes back to the humanistic perspective on organizations (Adams, 1992). It mainly draws on the idea that employees “should be able to participate in decisions which critically affect their conditions of employment” (Adams, 1992, p. 19). One of the characteristics that have been featured as related to workplace democracy is that this term represents a relative category (Stohl and Cheney, 2001). First and foremost, workplace democracy is considered a term that expresses an “alternative” way to manage and structure organizations in contrast to the traditional and thus hierarchical ways of organizing work.

Initially, there was a shared understanding that workplace democracy is characterized by non-hierarchical modes of management, private collective ownership, and democratic decision-making (Diefenbach, 2020). Similarly, Battilana et al. (2018) pointed out that different positions share the idea of workplace democracy as an organizational model that “involves (a) a broad diffusion of decision rights and (b) an organizational culture that entails some form of commitment to integrate individual perspectives with that of the broader organization; and finally, (c) in some cases, a broad diffusion of ownership rights.”

A more thorough look reveals that there is a wide array of heterogeneous organizational practices that are subsumed to workplace democracy with the result that its operationalization becomes a particularly challenging issue. In the conceptual considerations, different forms and areas of workplace democracy are discerned. Accordingly, Marchington and Wilkinson (2005) differentiated between (a) direct communication (e.g., face-to-face or written communication), (b) participation in decision-making and problem-solving (e.g., work circles, quality circles, health and project circles, and organizational systems for suggestions and complaints), (c) representative participation (e.g., representatives as elected by employees like works councils, trade unions, and collective bargaining), and (d) financial participation (e.g., profit-sharing, bonuses or stock options, and employee-owned companies).

One popular way to bring some differentiation into the operationalization of workplace democracy is to discern several degrees of it along the so-called “escalator of employee participation”—a metaphor that expresses various levels of participation provided to employees. the lowest level is represented by “information and communication to employees,” for example, providing selected business information to employees; it is followed by steadily rising degrees of participation at work, like “consultation,” for example, managers being formally or informally consulted by employees, and “co-determination,” which means that employees are taking part in strategic decision-making. “Employee control,” for example, employees making strategic decisions, represents the highest level of organizational participation (Wilkinson et al., 2010).

To sum up, workplace democracy remains an “umbrella concept” that encompasses different and highly heterogeneous models of decision-making structures as well as modes of organizational ownership. There are good reasons to assume that each form of workplace democracy can exert different influences on organizations and their members, including their skills and attitudes. Pateman (1970) pointed out in her work that different forms of workplace democracy assumingly lead to quite different effects in terms of political efficacy and participation skills to be learned. For example, representative forms of workplace democracy, Pateman argued, potentially have the lowest effect. Similarly, Greenberg et al. (1996) proposed that the research should go “beyond simple political spillover” and take into account different forms of workplace democracy. The scholars have also stressed that the effects of direct and representative workplace democracy should be considered separately.

As Adman (2008) and Kim (2021) pointed out, empirical studies till now fail to consider a full range of different types and levels of workplace democracy. There is also a lack of thorough consideration of formal and informal ways of workplace democracy. In the majority of the considered research, workplace democracy is measured as perceived autonomy at work in terms of being free to take part in decision-making and being able to change working methods or to decide when to start and to stop working (e.g., Elden, 1981; Peterson, 1992; Spreitzer, 2007; Budd et al., 2018; Timming and Summers, 2020). Although of crucial importance, perceived work autonomy only insufficiently covers structurally embedded modes of workplace democracy, like face-to-face participation or participation through works councils. There are only a few studies where structural types of workplace democracy are considered explicitly, with Weber et al. (2008) providing one of the rare examples of how institutional formats of workplace democracy could be operationalized. Based on such instances of workplace democracy, like the existence of works councils or similar collective decision-making structures, the authors distinguish seven levels of workplace democracy and provide a type-based index of workplace democracy for companies. Some authors (e.g., Pircher Verdorfer et al., 2013; Tak, 2017) have tended to tackle the issue of the structural side of workplace democracy while contrasting cooperatives and employee-owned companies with conventional firms, assuming that the former, in general, provide more democratic work settings than the latter. However, this implicit assumption has to be scrutinized since research on cooperatives demonstrates a huge diversity of cooperatives and shows that cooperatives are not necessarily democratic organizations since their aims, values, and ways of organizational decision-making are highly varying and oligarchic tendencies are one of the essential struggles in cooperatives (e.g., Varman and Chakrabarti, 2004; Heras-Saizarbitoria, 2014; Jaumier, 2017).

Similar criticisms have been raised in regard to the measurement of *political participation as the dependent variable*. A wide range of variables has been used as indicators of political participation. Several studies (e.g., Peterson, 1992; Sobel, 1993; Adman, 2008) used a summative index that consists of several indications of political participation, including highly heterogeneous variables as interest in politics, voting, membership of a political party, campaigning, and community activities. A critical consideration of potential discrepancies between these separate indicators is still lacking, although results from several studies pointed to the importance of considering these indicators in a differentiated way since different forms of political participation relate differently to perceived workplace democracy (e.g., Sobel, 1993; Greenberg et al., 1996). Another measuring approach is based on using rather general indicators, such as civic behavior, solidarity, or moral and pro-community orientation (e.g., Weber et al., 2008; Lopes et al., 2014). Although of high value, these variables are quite distal to political participation in terms of taking part in political decisions. To sum it up, political participation as a dependent variable has been operationalized in previous research either in a too wide or too strict sense, with both ways having far-reaching consequences for the validity of empirical findings. The empirical evidence is considerably threatened by the resulting inconclusiveness of operationalization and by lacking consideration of differences between included foci of political participation (Kim, 2021).

## Spillover thesis: Conceptual issues

In addition to the measurement-based limitations of the previous scholarship regarding the spillover thesis as discussed earlier, there is a number of conceptual limitations and unresolved questions too. As conceptual and empirical issues are strongly interlinked, a one-sided discussion regarding the democratic spillover and lacking solid empirical proof are the results of it. Although most of the conceptual questions are not new since they have been raised in previous research, the considerations until now have remained highly fragmented. In the following text, I provide a more systematic overview of the most relevant conceptual limitations and discuss them in more detail.

### Heterogeneous mechanisms of democratic spillover

The complexity of the argument by Pateman and, at the same time, its vagueness have led to different reinterpretations of it. The basic assumption, as shared by all these reinterpretations, is the idea of formal similarity between political and workplace realms. Both domains are assumed to be alike in that they share authority structures and main processes of decision-making (Greenberg, 2008), with the result that the potential transfer of participation experiences between the workplace settings and political settings could be assumed as high. When it comes to concrete reasoning of what constitutes the democratic spillover, an array of slightly different reconsiderations of Pateman's idea can be found in the research that has been conducted in the field. In his literature review, Greenberg (2008) pointed out at least three subsequent theses that have been derived by scholars from the original work by Pateman to undergird the nexus between workplace democracy and political participation. Each of these theses represents slightly different underlying psychological mechanisms in explaining how and why workplace democracy could have an impact on political participation. The theses are as follows:

1) *The thesis of increased efficacy* states that the sense of personal efficacy achieved by participating in one realm can be carried into other institutions. This is the main argument as proposed by Pateman (1970), who argued that when people are given opportunities to participate in their workplace context, they gain personal efficacy, which can lead to stronger or more effective participation in political processes.

2) *The thesis of the increased sense of commonality* suggests that opportunities to take part in decision-making regarding one's work, to discuss relevant issues with colleagues, and to reach an agreement lead to an increased social orientation of individuals. Such circumstances encourage people to deal with different positions and require that diverse interests are considered. Individuals become familiar with going beyond their personal interests and taking the needs of others more into account. Thus, this thesis claims for changes in interests and motives of behaving when having an opportunity to participate in decision-making at own workplace.

3) *The thesis of increased democratic skills* proposes that democratic workplaces with factual participation opportunities equip employees with relevant skills that are needed in any democratic setting and could be transferred beyond workplaces. According to Greenberg (2008), these skills include rhetorical competencies, such as speaking publicly, or organizational competencies, like moderating meetings and discussions.

The last thesis has currently received scholarly attention, for example, the study conducted by Summers and Chillas (2021) on employee-owned companies. The authors suppose that workplace democracy, for instance, concretely firm ownership by employees, supports individual democratic skills and competencies. Based on results from a qualitative study, Summers and Chillas (2021) further differentiated between two sets of democratic skills: First, there are democratic skills related to economic issues. The authors call them “skills in *economic* democracy.” These skills represent business owner skills, like financial literacy, business planning, and considering strategic issues of the business. Second, some skills directly link to *democratic* questions and give more emphasis to democracy and democratic functions, like building egalitarian relationships, achieving collective aims, and expressing opinions, but also socio-emotional skills, like empathy, caring, and regard for others. The authors call those “economic democracy skills.” They argue that these skills are the results of democratic workplace settings as they are often given in employee-owned companies and as they are required by a democratic society, just like Pateman (1970) claimed. Summers and Chillas (2021) considered these two sets of skills in economic democracy as a crucial factor for answering the question of organizational performance in the case of employee-owned companies.

As Pateman (1970) considered political efficacy as the most crucial moderator between workplace democracy and political participation, the issue of remaining potential moderators also deserves to be raised in regard to the underlying mechanisms of democratic spillover. This particular issue has received only scarce scholarly attention until now and still lacks a systematic analysis. For example, Greenberg et al. (1996) argued in favor of the economic situation of the firm (i.e., being in trouble or not) as a potentially important moderator for the relationship in question.

Notwithstanding the considerations provided, there are several unresolved issues related to the different mechanisms of democratic spillover. First, there remains to be clarified how different mechanisms of democratic spillover are interlinked, for example, whether acquiring political efficacy corresponds with gaining democratic skills and an increased sense of commonality. Second, a robust explanation of how and under which circumstances respective skills are acquired and under which conditions they are not is still lacking. Here, an in-depth consideration of potential moderators could be of particular importance when revealing the explanatory processes of democratic spillover. It is also to be clarified which skills acquired in the course of workplace democracy are related to political participation and which are not and thus when the mechanisms mentioned work and when they do not and why. It becomes clear that these issues need additional and substantial conceptual attempts in order to be resolved and to provide a more precise idea concerning the questions of “how” and “what” of democratic spillover.

### Alternative explanations

Empirical scholars have repeatedly pointed out that the relationship between workplace democracy and political participation is probably more complex than assumed by Pateman. The argument of democratic socialization and political efficiency through workplace democracy, as conveyed by Pateman, deserves to be scrutinized. Beyond the mere positively moderated spillover, different types of links between organizational and political democracy can be identified.

One alternative kind of relationship that has received only scarce attention by researchers until now is *reverse causality* as the democratic spillover can be thought of in the reversed direction: as a spillover from the political realm to the workplace settings. It means that not workplace democracy leads to political participation, but vice versa is the case: because of political participation, citizens are more prone to take part in democratic measures at work (or to expect such opportunities). It could thus be assumed that experience made in political settings can affect workplace behavior and expectations regarding job autonomy and decision-making at the workplace. Nevertheless, this relationship could potentially be moderated by political efficacy. Early support for the thesis of reversed causality was provided by Witte (1980), who stated that employees taking part in participation programs are significantly more likely to have participation experiences in the political realm. In a similar vein, the study by Pineiro Harnecker (2009) in the field of Venezuelan worker cooperatives has yielded results that at least indirectly point to the reverse causality. The author argued that when cooperative members have experiences in community participation, these cooperatives develop a higher degree of social consciousness and solidarity with local communities. Accordingly, Kim (2021) suggested performing research that would be able to rule out alternative explanations, like reverse causality. From a conceptual perspective, a broader minded and thorough analysis of possible relationships between workplace-based and political participation, as well as of political learning processes, in general, would be more fruitful than just ruling it out.

The next alternative way of framing the nexus between the workplace and politics is *the negative spillover*: It could be assumed that under certain conditions, workplace democracy leads to lesser political activity, like lower participation in elections. In the debate on democratic spillover, this issue has been considered only by a few scholars till now. Schweizer (1995) and Carter (2006) pointed to a potentially negative spillover from workplace democracy to the political realm because of structural differences. The authors pointed to the fact that workplace democracy and political efficacy as resulting from it are mostly nurtured by direct participation at the workplace; in contrast to this, political democratic structures are mainly based on representative formats. This structural difference may discourage employees who are familiar with taking part in workplace-oriented decisions from participating in the political realm.

In addition, we should take the complexity of potential effects into account since in the context of workplace democracy, employees gain different experiences, with some of them contributing to the sense of efficacy and others not. For example, in the case of pseudo-participation, where employees are not provided with serious opportunities to take part in decision-making and are instead increasingly controlled by participative measures (Mccarthy, 1989), the development of political effectiveness is rather less likely. Instead, in this case, political disappointment, powerlessness, or senselessness could be expected. Political disappointment could also be the case when workplace democracy is seriously intended, as soon as democratic undertakings are experienced as particularly time- and resource-consuming, often fraught with conflicts.

Negative spillover also relates to increased political participation not because of workplace democracy but because of the absence of it. For example, Geurkink et al. (2020) showed in their study that suppressing the voice of employees triggers their political participation. Supporting the same thread of argument, Lup (2022) provided results indicating that there is a path between discrimination experienced by employees at the workplace and their subsequent political activity.

To sum it up, positive spillover from the workplace to politics, as argued by Pateman, seems to be an ideal and not a regular case. Different (positive as well as negative) effects on political participation from successfully practiced workplace democracy as well as from failed workplace democracy (e.g., pseudo-participation) are possible. This requires a more differentiated consideration of the underlying mechanisms than was the case in previous studies.

### Dynamic nature of democratic spillover

One additional issue that underscores the fact that the relationship between workplace democracy and political participation is a non-direct relationship refers to dynamic and time-based processes. As Adman (2008) has demonstrated in his work, cross-sectional results show some significant associations between workplace and political participation. However, these links prove insignificant in long-term consideration. It remains open to what might be the role of time and time lag in this respect. Our knowledge is still limited on how long it takes to “learn” democracy and participation at the workplace and whether democratic spillover could be assumed immediately after first encounters with workplace democracy or rather with a considerable time lag. When dealing with these issues, research on time in organizational socialization processes (e.g., Ashforth, 2012) might be of particular help.

The time dimension implies that the spillover effect may follow a dynamic trajectory and leads us to think about a stage-based relationship between workplace democracy and political participation as one potentially helpful way to explain it. That would mean that this relationship should be considered a developing relationship, as framed by certain dynamic patterns. As workplace democracy is a process with its ups and downs, different stages can be assumed, such as the “first encounter,” “euphoric stage,” “stage of disillusionment,” and “stage of realistic agency,” with each stage potentially bearing different effects regarding political participation.

### Institutional settings to be considered

One additional issue that deserves explicit attention, but has received only cursory consideration in the previous studies, is the institutional layer of the democratic spillover. In previous research, country-related issues, if any, have received rather formal attention by treating them merely as a control variable, although the countries studied have a quite specific historical legacy and present agenda in regard to workplace democracy, be it the law of co-determination in Germany or the self-management in the Balkan countries. A thorough analysis of local beliefs and formal institutions regarding workplace democracy and political participation is mainly absent. The result is that our knowledge regarding the effects of workplace democracy is particularly generic and cursory as long as local country- and company-specific institutional settings remain disregarded.

Previous research has also mainly ignored any interconnections between different institutional levels of democratic socialization, including families and the educational sector. Thus, our knowledge about how experiences from the education system relate to workplace democracy is limited. We still do not know whether styles of primary and secondary socialization (e.g., education in families, schools, and high schools) cumulate or rather collate with tertiary socialization (e.g., workplaces). We also do not know what happens when education and the workplace stand in a sharp conflict, as might be the case when democratically socialized young employees are confronted with hierarchic authority structures at the workplace and lacking elements of participation and workplace democracy. We also do not know whether these collusions of socialization lead to a negative or rather positive spillover regarding political behavior.

## Future research prospects

Previous sections indicate numerous ways prospective research on democratic spillover needs to consider in order to achieve more consistent and comprehensive results. Ideally, conceptual and empirical issues should simultaneously be taken into account as unresolved conceptual shortcomings cannot be counterbalanced without empirical and method-based progress. In the following paragraphs, I indicate general avenues that may pave the way for prospective research on democratic spillover.

### Extended theoretical framework

Although the sheer elegance of Pateman's (1970) argument is beckoning, the analysis provided earlier makes clear that the original model, as proposed by Pateman, is too vague and needs theoretical amendments or theoretical syntheses with other theories or frameworks in order to adequately explain underlying processes and to guide respective empirical undertakings.

A potentially fruitful approach is the concept of *psychological ownership* (Pierce et al., 2001, 2004; Pierce and Jussila, 2010). drawing on the idea of collective ownership, the concept of psychological ownership points to, for example, a higher self-efficacy (Bandura, 2006), a sense of belonging, the perceived responsibility, and identification with a certain social group (e.g., Pierce et al., 2001; Avey et al., 2009) as mechanisms that explain why workplace democracy could have positive effects onto organizational behavior of individuals. Whether psychological ownership could be fruitful when explaining democratic spillover and how these explanations could look like might be valuable undertakings for prospective studies. In particular, the arguments of psychological ownership might be useful when tackling the issue of different mechanisms of democratic spillover, as considered in heterogeneous mechanisms of democratic spillover. When trying to consolidate previous heterogeneous assumptions regarding the democratic spillover, mechanisms of psychological ownership like the sense of belonging or the perceived responsibility might be relevant explanations for when workplace democracy spills over to social and political activities of employees beyond their workplaces and when not.

The concept of psychological ownership could also be helpful when dealing with non-participation, both in the case of employees at the workplace and citizens in the political realm. This issue has been neglected until now in the field of democratic spillover since scholars have implicitly assumed that participation is the regular case, and non-participation is just a marginal sub-topic. Looking for when and why workers or citizens refuse to take part in decision-making, despite manifold opportunities provided (Mccarthy, 1989), is an issue that deserves in-depth theoretical considerations. Psychological ownership could be one potential point of departure since it allows raising such questions as when self-efficacy and sense of belonging do not evolve and what consequences it could have for democratic engagement.

What deserves explicit consideration too are new issues emerging from current management practices, for example, agile work methods. Although claimed to represent modern forms of workplace democracy (e.g., Boes et al., 2018; Sauer et al., 2021), the issues of workplace agility are still barely covered by the previous research from the democratic spillover. Once again, the concept of psychological ownership could be helpful in analyzing and explaining various effects of agile working on employees, including the consequences of workplace agility on societal and political participation.

Although, Pateman (1970) has argued in favor of the educative effects of workplace democracy in regard to political democracy, she remains quite generic in explaining it. The consequence is that alternative explanations, like reverse causality or negative spillover, should be taken into account, as discussed in alternative explanations. A potential theoretical underpinning for the original argument as brought forward by pateman could be provided by the concept of *moral development in organizations* (Hannah et al., 2011). The concept proposed by Hannah et al. (2011) links moral sensitivity to moral action, just like the democratic spillover relates employees' experiences at the workplace to the increased sensitivity toward democratic issues and actions (e.g., political activity). Hence, the concept of moral development in organizations could be a promising theoretical frame when explaining whether and under which conditions democratic learning is taking place in working settings and leads to political activities of employees, as proposed by Pateman (1970).

When it comes to an insufficient consideration of institutional settings of democratic spillover, as mentioned in institutional settings to be considered, the integration of arguments from the theoretical perspective of *the sociological neo-institutionalism* (e.g., Scott, 1995) might be of particular relevance. The neo-institutionalism points to the relevance of taken-for-granted beliefs, which constitute the legitimacy of certain issues or practices. Accordingly, we could suppose that broader institutional settings, like implicit cultural norms in a society, significantly frame the status of workplace democracy as legitimate and acceptable (or not) and can support or impede democratic spillover. In a narrower sense, country-specific, local institutional settings, such as industrial laws, could also be assumed of particular importance when explaining whether and what kind of experiences workers are making with workplace democracy and how these experiences affect the political attitudes and behavior of employees. Especially, the research on legitimacy (e.g., Suchman, 1995) or on institutional logics considered as cultural frames of reference that condition sensemaking and actions of individuals (e.g., Thornton and Ocasio, 2008; Cornelissen and Werner, 2014) could provide suitable conceptual complement when explaining when and why workplace democracy is considered as legitimate and when political participation by employees becomes taken for granted. By drawing on the theoretical perspective of the neo-institutionalism, the debate regarding democratic spillover could receive important conceptual stimulation for developing a more comprehensive theoretical model and for generating context-sensitive knowledge on democratic spillover.

### Qualitative method approaches

Previous empirical research on democratic spillover is dominated by quantitative approaches, as shown in Table 1. Future studies should include much more diverse approaches and complement dominating quantitative survey-based studies with qualitative inquiries, based, for example, on long-term observations, case studies, or narrative interviews. Especially, when trying to empirically approach the dynamic nature of democratic spillover, as argued in Dynamic nature of democratic spillover, scholars should take qualitative methods much more into account than previously. Long-term interviews, case studies, or ethnographical approaches would be able to provide a much higher degree of context-sensitive and comparative material about the societal effects of workplace democracy than this is the case in most quantitative undertakings. For example, research drawing on contrasting cases that include companies with established elements of workplace democracy as well as firms with absent or failed workplace democracy might be of particular value here when trying to determine different effects on political participation. Numerous examples of qualitative undertakings based on case studies of cooperatives might serve as helpful references in terms of method, like the analysis of cooperatives in the United States by Rothschild and Whitt (1986), a comparative study between John Lewis Partnership in the United Kingdom and Eroski in Spain as conducted by Storey et al. (2014), or a case study-based typology of participation patterns in German cooperatives by Hühn et al. (2021).

When using qualitative approaches, more attention could also be given to country-based studies that allow an in-depth analysis of local institutional settings as well as qualitative comparisons between selected countries. By doing this, a deeper understanding of the institutional and country-specific influences would be possible, which frame the relationship between organizational and political democracy. A country-sensitive in-depth analysis, as well as systematic qualitative cross-country comparisons, would be able to provide fruitful insights into the debate surrounding workplace democracy. Furthermore, in difference to individual variables that mainly stood at the focus in previous quantitative undertakings, much more attention could receive institutions of industrial relations and employee representations, as well as varieties of capitalism, including country- and region-specific patterns of democracy. Given the assumed relevance of regional and historical legacies in the case of democratic spillover, specific regions could be purposefully addressed in such empirical undertakings. One example would be the Central and Eastern European countries and thus former European socialist countries. This region represents a particularly beneficial research context in terms of workplace democracy and its transformations due to specific historical legacies like workers' self-management in the former Yugoslavia or due to current democratic struggles and oligarchic tendencies, like in Poland or Hungary.

## Summary, conclusion, and practical implications

In her seminal work on how workplace settings might be related to the political participation of citizens, Pateman (1970, p. 66) highlighted that industry plays a central role “in the democratic socialization process.” By pointing to the political efficacy as stemming from democratic encounters at the workplace, Pateman provided a psychological argument for why working life and democratic measures provided in industrial and workplace settings are of crucial relevance for political democracy in societies. Considering current political upheavals and increased populist tendencies in the EU, the United States, and elsewhere, the argument by Pateman proves even more important at current times than at the time of its original publication as it provides a path for how democratic societies could be strengthened.

Nevertheless, it should be stated that research that has been made on the democratic spillover represents an academic niche. Moreover, existing empirical findings are inconsistent and provide mixed evidence for the original thesis. Following, Greenberg et al. (1996), we have to state that the spillover effect is not suited to argue for the strength of workplace democracy as there is no definitive evidence for this. The debate suffers several methodological and conceptual limitations that hamper yielding consistent results and that future research needs to tackle. From the conceptual point of view, these limitations include, in particular, the heterogeneity of potential spillover mechanisms, a lacking consideration of alternative explanations (e.g., reversed causality or negative spillover), an insufficient analysis of the potentially dynamic nature of the relationship, and a lacking in-depth consideration of institutional settings, like country-based specifics in relation to workplace democracy and formats of political participation. By scrutinizing existing research and by identifying its main shortcomings from the conceptual perspective as well as by showing some possible paths for prospective research, the present article provides a relevant theoretical contribution to the debate on democratic spillover.

The argument by Pateman (1970) should be yet considered a relevant point of departure, but not the end of the conceptual debate. The conceptual progress of this debate remains particularly challenging because of the issue of multiple determinants of political participation: workplace democracy represents one potentially relevant, but not an exclusive, reason for the political participation of employees. Moreover, as existing research still lacks a robust theoretical explanation of the relationship between workplace democracy and its political effects, there is an urgent need for concise and fine-grained conceptual developments that would tackle the question of how and when workplace democracy leads to political learning or political efficacy (and when not). Among others, the processes related to workplace democracy, including the absence and failure of workplace democracy, require more comprehensive analyses that would be able to cover the complexity of the issue and reinvigorate the debate on democratic spillover.

In particular, interdisciplinary work approaches are needed to intersect different fields, as touched upon by democratic spillover. In order to establish a solid conceptual and empirical scholarship on democratic spillover and to appropriately cover the complexity of related issues and counterbalance the one-sidedness of previous conceptual considerations, undertakings are needed where scholars from political sciences, organizational sociology, psychology, pedagogy, management studies, and industrial relations take part.

The present analysis primarily deals with research that has been carried out in the field of democratic spillover with the aim to critically reflect research-based assumptions and results yielded and does not deal with practical techniques, tools, or training programs in this field. Nevertheless, the results obtained have a lot to do with the work practice. Thus, we end this article with practical implications. The primary practical implication of this article is sensitizing: it matters whether workplaces include democratic elements or not. It matters surely in terms of individual measures, like job satisfaction and job commitment; it matters in terms of organizational effectiveness, like organizational performance and innovativeness. Potentially, yet not consistently confirmed in empirical studies, it matters in terms of the political participation of employees. Until now, there is no clear evidence that workplace democracy leads to increased political participation of employees, but there is also no clear support that it does not. “The long arm of the job” in mind we have to conclude that industrial companies and organizations are not insular elements of the economy; as providers of workplaces for the majority of societal members, companies and industries represent one potentially relevant dimension of sustaining democratic systems of respective societies. Managers should have this in mind when designing workplaces and jobs when considering communication processes and decision-making in their organizations. From this perspective, the “democratic dimension” should be considered a part of social responsibility in organizations since it represents a suitable way to contribute to democratic societies by establishing workplaces that could socialize employees as democratic citizens.

## Data availability statement

The original contributions presented in the study are included in the article/supplementary material, further inquiries can be directed to the corresponding author.

## Author contributions

The author confirms being the sole contributor of this work and has approved it for publication.
